# Visualizing Presynaptic Active Zones and Synaptic Vesicles

**DOI:** 10.3389/fnsyn.2022.901341

**Published:** 2022-05-18

**Authors:** Manfred Heckmann, Martin Pauli

**Affiliations:** Department of Neurophysiology, Institute for Physiology, Julius-Maximilians-University Würzburg, Würzburg, Germany

**Keywords:** active zone, depression, facilitation, plasticity, potentiation, synapse

## Abstract

The presynaptic active zone (AZ) of chemical synapses is a highly dynamic compartment where synaptic vesicle fusion and neurotransmitter release take place. During evolution the AZ was optimized for speed, accuracy, and reliability of chemical synaptic transmission in combination with miniaturization and plasticity. Single-molecule localization microscopy (SMLM) offers nanometer spatial resolution as well as information about copy number, localization, and orientation of proteins of interest in AZs. This type of imaging allows quantifications of activity dependent AZ reorganizations, e.g., in the context of presynaptic homeostatic potentiation. In combination with high-pressure freezing and optogenetic or electrical stimulation AZs can be imaged with millisecond temporal resolution during synaptic activity. Therefore SMLM allows the determination of key parameters in the complex spatial environment of AZs, necessary for next generation simulations of chemical synapses with realistic protein arrangements.

## Introduction

### Presynaptic Active Zones and Synaptic Vesicles

Neurotransmitter release at presynaptic active zones (AZs) of chemical synapses is currently investigated intensely and was recently reviewed from various perspectives (Brunger et al., 2018; Silva et al., 2021; Werner et al., 2021; Rizo, 2022; Wichmann and Kuner, 2022). Since the term AZ was coined in the last century (Tsuji, 2006) substantial progress occurred. As in any research field, quite obviously, specific techniques such as electron microscopy (EM), molecular genetics, structural biology, and patch clamp electrophysiology provide complementary data. In view of decades of research, it remains fascinating to witness how preparations and techniques for AZ research are continuously refined and more precise quantitative information accumulates.

Electron microscopy of AZs continues to provide critical insights, as for example was recently shown for structural transitions during synaptic vesicle (SV) priming (Grushin et al., 2022; Lichter et al., 2022). Evaluating the dimensions of electron dense material below docked SVs can provide information how the chaperone Munc13, synaptotagmin, SNARE complexes, and complexins are assembled (Rothman et al., 2017; Zhou et al., 2017). Furthermore, the combination of EM and high pressure freezing (HPF) with optical stimulation, aptly termed flash-and-freeze (Watanabe et al., 2013a,b; Chang et al., 2018; Borges-Merjane et al., 2020; Imig et al., 2020; Vandael et al., 2020), or electrical stimulation (zap-and-freeze, Kusick et al., 2020; Li et al., 2021; Vevea et al., 2021), brings certainly more than just a breath of fresh air to the field. Flash-and-freeze or zap-and-freeze provide millisecond resolution for activity dependent imaging of SVs in AZs. Although it is difficult to make predictions, especially about the future, it is probably safe to expect further substantial progress along this line of investigation.

We focus on yet another technique for super-resolution of SVs and AZs, namely localization microscopy in the form of *direct* stochastic optical reconstruction microscopy (*d*STORM; van de Linde et al., 2011). *d*STORM offers protein specificity and in ideal cases spatial resolutions of several nanometers. This was nicely demonstrated for nuclear pore complexes (Löschberger et al., 2012, 2014), which despite inherent flexibility (Schuller et al., 2021), are used as reference standards for quantitative super-resolution microscopy (Thevathasan et al., 2019). In the following we will: (1) present paradigmatic *d*STORM data for two types of synapses, (2) discuss *d*STORM of activity dependent AZ rearrangements, (3) return to the above-mentioned combination of HPF with either optical or electrical stimulation and will argue how *d*STORM of AZs may profit from HPF with stimulation, and (4) end by touching the topic how a realistic ultrastructure can be achieved in next generation simulations of SVs in AZs.

## Results and Discussion

### Distinct Presynaptic Active Zones

**FIGURE 1:**
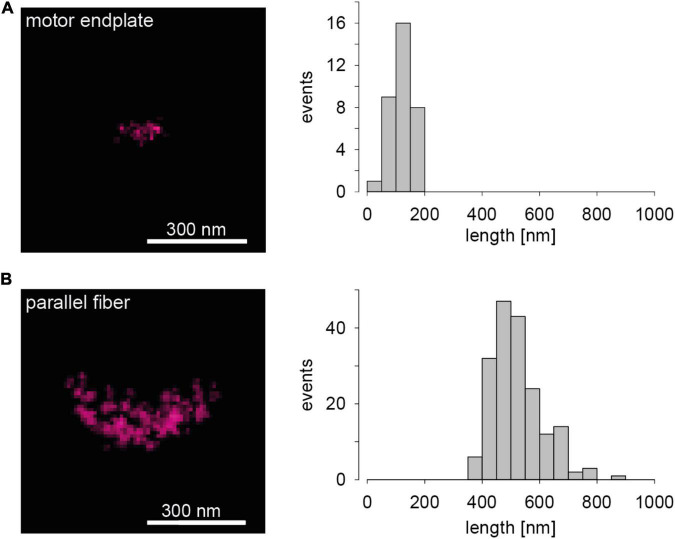
Presynaptic Bassoon AZ scaffold in two murine synaptic connections. **(A)** The left panel shows a representative 2D one-color *d*STORM image of a Bassoon cluster in a mouse motor endplate of a *Levator auris* muscle preparation. On the right a histogram for Bassoon cluster length is displayed (*n* = 34 AZs, length = 120 ± 35 nm, mean ± SD). **(B)** The left panel shows a *d*STORM image of a Bassoon cluster in a parallel fiber to Purkinje neuron synapse in a sagittal cryosection of a mouse cerebellar vermis. On the right again, a histogram for Bassoon cluster length is shown (*n* = 184 AZs, length = 520 ± 88 nm).

Images of the abundant presynaptic AZ scaffolding protein Bassoon (Gundelfinger et al., 2016) in two chemical synapses of a mouse serve to introduce *d*STORM images of AZs. The left panel in Figure 1A shows a representative Bassoon cluster in an endplate of a neuromuscular junction (NMJ). The left panel in Figure 1B illustrates a representative Bassoon cluster in a cerebellar parallel fiber (PF). The right panels in Figure 1 show histograms of cluster length in the two preparations. The experimental imaging was essentially performed as described earlier for 2D *d*STORM in 1 μm thick tissue sections (Pauli et al., 2021). The substantial difference in Bassoon cluster length in Figures 1A,B is absolutely obvious and fits quite well to EM data of AZ dimensions in the two preparations (Nagwaney et al., 2009; Indriati et al., 2013). Since protein amount can be measured with *d*STORM by quantifying the fluorescence signal, more precisely the number of localizations (Löschberger et al., 2012, 2014; Ehmann et al., 2014), the two images in the left panels of Figure 1 also reveal that the Bassoon content of endplate AZs is certainly much lower than that of PF AZs. Significant differences of Bassoon cluster length, localizations per cluster (Bassoon content) and cluster volumes were also measured recently for three types of hippocampal principal neurons, even within single tissue sections, using *d*STORM (Figure 4 in Pauli et al., 2021). In principle, protein specific quantitative results with *d*STORM for AZs are similar to data obtained by freeze-fracture replica immunogold labeling (Hagiwara et al., 2005; Éltes et al., 2017; Mrestani et al., 2021; Pauli et al., 2021). Figure 1 serves as illustration that it is possible to measure the amount and the distribution of proteins of interest in AZs with *d*STORM.

### Activity Dependent Rearrangements

While protein distribution measurements with scale bars of 300 nm (Figure 1) are valuable, another level is reached by more detailed evaluations of the fine structure of *d*STORM data. For the *Drosophila* NMJ, it was reported that the AZ cytomatrix is composed of units containing approximately 137 copies of Bruchpilot (Brp) protein, three quarters of which are organized into about 15 heptameric clusters (Ehmann et al., 2014). Bassoon and Brp are often used as AZ markers, since they are relatively abundant AZ proteins.

**FIGURE 2:**
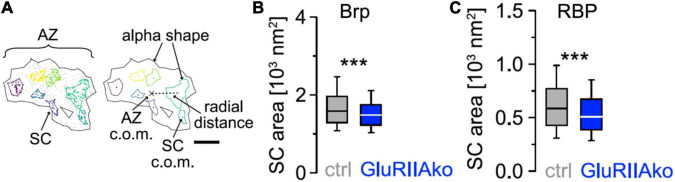
Compaction of presynaptic AZ SCs **(A)** HDBSCAN for SC detection applied to an AZ of a wild-type Ib bouton of a *Drosophila melanogaster* neuromuscular junction stained for Brp*^NC82^*. Black lines indicate alpha shapes used for AZ area quantification. Left: colored Brp SCs surrounded by colored lines indicating alpha shapes. Gray dots represent unclustered localizations. Right: centers of mass (c.o.m.) of the AZ (cross) and of SCs (colored dots) are indicated. A dashed line shows the Euclidean distance between the AZ c.o.m. and an SC c.o.m., referred to as radial distance. Scale bar 100 nm. **(B)** Chronic PHP (GluRIIAko) decreases Brp SC area. Scatterplots of Ib AZs from control (ctrl) and GluRIIAko animals. **(C)** Compaction of RBP SCs at GluRIIAko AZs. Scatterplots of Ib AZs from control (ctrl) and GluRIIAko animals. Reprinted from Mrestani et al. (2021). ***p < 0.001.

More recently, hierarchical density-based spatial clustering of applications with noise (HDBSCAN) was used to study Brp distribution and AZ plasticity during presynaptic homeostatic potentiation (PHP; Mrestani et al., 2021; Figure 2). Compaction of individual AZs was found in acute philanthotoxin-induced and chronic genetically induced PHP but Brp protein copy numbers did not change. Compaction occurs even at the level of Brp subclusters (SCs), which additionally move toward AZ centers. Furthermore, compaction happens also within SCs of Rab3 interacting molecule-binding protein (RBP), another AZ protein closer to the membrane (Mrestani et al., 2021). Brp SCs in wild type have average diameters of about 46 nm, compared to a diameter of about 27 nm for RBP SCs (Mrestani et al., 2021, note scale difference in Figures 2B,C). Furthermore, RBP SCs contain only about 1/3 of the localizations of Brp SCs, and RBP is thus less abundant in AZs (Mrestani et al., 2021). However, other proteins, such as calcium channel subunits, are again substantially less abundant than RBP, but still readily detectable in AZs with *d*STORM and related techniques (Ehmann et al., 2015; Newman et al., 2022). AZ proteins can also be arranged in still smaller clusters with diameters well below the diameter of a typical SV, approaching the localization precision of about 6 nm in our *d*STORM images (Mrestani et al., 2021). It might thus be possible to obtain activity dependent *d*STORM protein maps, e.g., for presynaptic calcium channels or other less abundant AZ proteins.

### High Pressure Freezing, Stimulation, and *Direct* Stochastic Optical Reconstruction Microscopy of Active Zones

Facing activity dependent positional changes of AZ proteins with *d*STORM, we are led to ask: What could be gained by combining *d*STORM with flash- or zap-and-freeze? HPF alone eliminates artifacts commonly associated with classical chemical fixation. More fascinating is, that *d*STORM after flash- or zap-and-freeze should yield AZ protein maps with millisecond temporal resolution after synaptic stimulations. While it is not entirely clear how much spatiotemporal protein changes we will find with such investigations, it is instructive to speculate briefly about such experiments. Protein arrangements of SVs change during fusion (Brunger et al., 2018; Rizo, 2022). Perhaps more subtle protein repositioning during SV fusion will remain invisible with currently achievable *d*STORM. However, larger protein displacements such as, e.g., the ones of complexin and Rab3 during SV fusion might be resolvable. Furthermore, other quantitative predictions, such as the hexameric arrangement of Munc13 below docked SVs, or the position of synaptotagmin on SVs (Rothman et al., 2017; Grushin et al., 2022) could be experimentally tested. We should probably move from whole mounts or currently used 1 μm thick tissue to thinner sections, for these experiments. A section thicknesses of 50 nm or below, and thus in the range of the diameter of individual SVs or even below, might be ideal. Perhaps even serial sections or correlative light and electron microscopy (CLEM) should be tested (Markert et al., 2016). Furthermore, beyond inspections of individual SVs it may be instructive to map the AZ scaffold further with millisecond resolution during the SV cycle with single-molecule localization microscopy (SMLM).

### Next Generation Simulations

Models of SVs and AZs should be built considering all available data and thus be reality based. With increasingly detailed ultrastructural data for SV and AZ proteins the mesoscale, bridging the atomic nanoscale and the cellular microscale, still remains largely invisible (Goodsell et al., 2020). This shortcoming can be addressed and we argued above how critical information regarding copy number, localization, and orientation of key SV and AZ proteins can be obtained with *d*STORM. It appears the time is ripe for further measurements of activity dependent protein rearrangements of SVs and AZs with SMLM. This might in turn set the stage for next generation simulations of chemical synapses with a more realistic protein nano-architecture.

## Data Availability Statement

The raw data supporting the conclusions of this article will be made available by the authors, without undue reservation.

## Ethics Statement

Ethical review and approval was not required for the animal study because according to German regulation tissue was taken from sacrificed animals.

## Author Contributions

MH and MP conceived, wrote, and revised the manuscript. Both authors approved the submitted version.

## Conflict of Interest

The authors declare that the research was conducted in the absence of any commercial or financial relationships that could be construed as a potential conflict of interest.

## Publisher’s Note

All claims expressed in this article are solely those of the authors and do not necessarily represent those of their affiliated organizations, or those of the publisher, the editors and the reviewers. Any product that may be evaluated in this article, or claim that may be made by its manufacturer, is not guaranteed or endorsed by the publisher.
